# Dosimetric and clinical validation of a high‐density bolus for superficial skin tumors treated with radiotherapy

**DOI:** 10.1002/acm2.70718

**Published:** 2026-07-29

**Authors:** Jonathan Dadoun, Ann Van Esch, Noemie Litrowski, Carine Lefrancois‐Clement, Erwan Luce, Alexis Thelie, Nassima Delhoumi, Andrea Feuillet, Nesrine Zemman, Romain Mallet, Renata Pereira, Laurent Martin, Paul Gangloff, Christophe Moure, Luc Ollivier, Paul Lesueur

**Affiliations:** ^1^ Radiotherapy Center Guillaume le Conquérant Le Havre France; ^2^ Quality Assurance Team in Radiotherapy Physics 7Sigma Tildonk Belgium; ^3^ Dermatology Department Groupe Hospitalier du Havre Le Havre France; ^4^ Head and Neck Surgery Department Hôpital Privé de l'Estuaire Le Havre France; ^5^ Maxillofacial Surgery Department Clinique des Ormeaux Le Havre France; ^6^ Radiation oncology department Institut de Cancérologie de l'Ouest Saint‐Herblain France; ^7^ ISTCT/CERVOxy Group UMR6030, GIP CYCERON Normandy University Unicaen Caen France

**Keywords:** Acuros XB, bolus, high‐density, radiotherapy, skin cancer

## Abstract

**Background:**

Accurate dose deposition in superficial radiotherapy remains challenging due to the inherent skin‐sparing effect of megavoltage photon beams and difficulties in ensuring bolus conformity.

**Purpose:**

This study aims to comprehensively evaluate the dosimetric characteristics of the room‐temperature‐malleable high‐density bolus, to evaluate the accuracy of dose calculation and to assess clinical outcomes in a clinical cohort of 55 patients.

**Materials and Methods:**

The dosimetric validation was performed through the use of dose measurements in a solid water phantom on top of which a 1.0 cm thick high‐densitybolus was positioned. Absolute depth dose measurements beyond the bolus were obtained by combining an absolute point dose (ion chamber) measurement at 1.0 cm depth beyond the bolus with relative depth dose measurements by using a microDiamond detector. Measurements were performed using 6 megavoltage (MV) flattening‐filter‐free (FFF) photon beams on an Halcyon and on a TrueBeam treatment unit. Obtained data were compared to dose calculations with both photon dose calculations available in the Eclipse treatment planning system (TPS): Analytical Anisotropic Algorithm (AAA) and Acuros XB (AXB). The impact of non‐conforming interfaces was assessed by introducing 0.5 to 2.0 cm thick air cavities between bolus and skin. Skin dose loss due to the air cavities was measured for static open fields and for dynamic sweeping gap fields. Clinical outcomes of 55 patients treated between October 2023 and March 2025 were retrospectively analyzed.

**Results:**

Depth dose measurements confirm that a 1.0 cm high‐density bolus was sufficient to effectively overcome the build‐up region of the 6FFF photon beam. The AXB dose calculation algorithm provided the best agreement between measurements and calculations. Air gaps between the bolus and the skin resulted in clinically relevant skin dose reductions, ranging from approximately 1% to 20%, depending on field size and gap thickness, especially for clinical target volumes < 5.0 cm, large (> 1.0 cm) air gaps, or the use of large static fields. Among the 55 included patients, acute dermatitis toxicity was predominantly Grade 1 (49%) or Grade 2 (47.0%), with a low incidence of severe adverse events (Grade III: 4 %, Grade IV: 0%).

**Conclusion:**

The high‐density bolus demonstrated satisfying dosimetric reliability, particularly when modeled with the Acuros XB algorithm. The bolus addressed the build‐up challenge inherent to MV photons, even in the presence of small air gaps, without increased toxicity in a large cohort of patients. This strategy represents a viable and accessible solution for ensuring accurate and conformal dose delivery in superficial tumors.

## 1 INTRODUCTION

The treatment of superficial skin tumors, including basal cell and squamous cell carcinomas, is a constant challenge in radiation‐oncology, mainly related to the need to ensure adequate delivery of the dose to the skin surface while taking into account the underlying anatomical irregularities.^1^ The inherent skin‐sparing effect of megavoltage photon beams dictates the use of a build‐up material (bolus) to ensure sufficient dose delivery to the superficial planning target volume (PTV). Historically, electron beams or conventional tissue‐equivalent boluses, such as Superflab (density ⋍1.02 g/cm3) were employed to manage the skin‐sparing effect characteristic of megavoltage photon beams.^2^

However, electron beams are associated with significant dosimetric uncertainties when encountering material heterogeneities, air‐tissue interfaces, geometric complexities, or factors that compromise calculation accuracy^3^ and homogeneous dose delivery. Modern linear accelerators such as Varian Halcyon© predominantly rely on advanced photon techniques like Volumetric Modulated Arc Therapy (VMAT), leading to a strategic shift towards photon‐based treatments for superficial lesions.^4^, ^5^

When using photon beams, the use of bolus materials is crucial to ensure dose build‐up and conformal dose distribution at the skin interface.^1^ Conventional soft bolus often fail to achieve optimal anatomical conformity, leading to unintended air gaps. When photon radiotherapy is used, Air gaps can reduce skin dose, with an amplitude depending on the air gap thickness and irradiation field size. For VMAT deliveries, air gaps compromise dose homogeneity and reproducibility.^1^, ^6^

**FIGURE 1:**
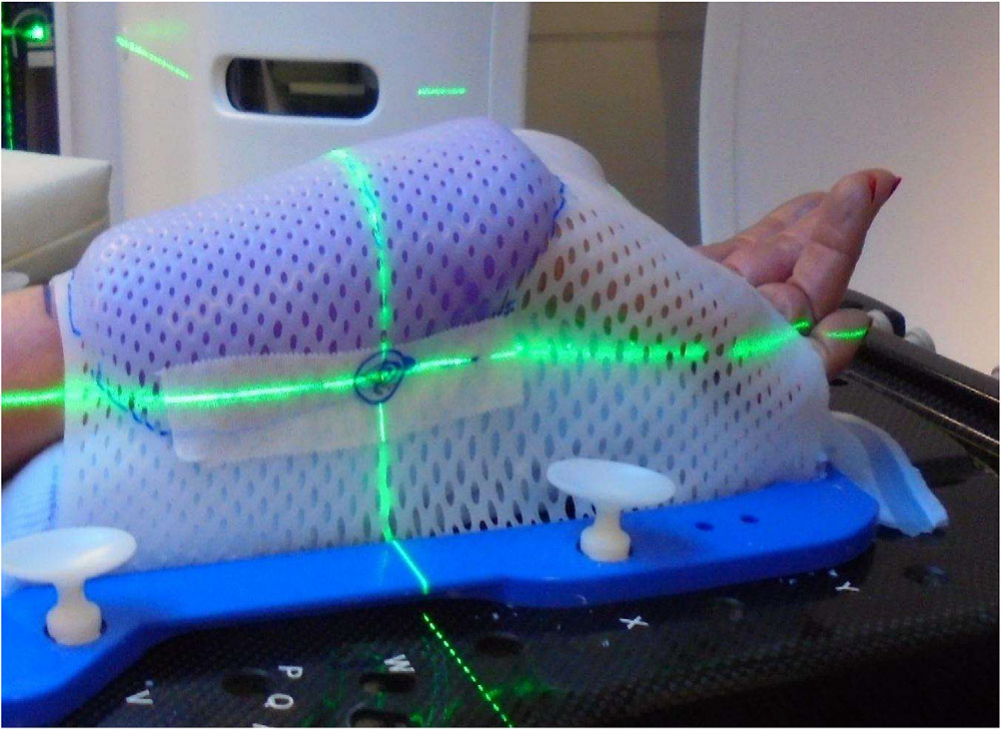
High‐density bolus positioned under a thermoplastic immobilization mask for treatment of right wrist skin cancer.

This innovative high‐density bolus (Figure 1) is malleable at room temperature, offering superior adaptation to complex contours (e.g., ear, nose) and it has a relatively high‐density of 1.59 g/cm3, comparable to cortical bone.^1^ The latter reduces the total bolus thickness required to overcome the build‐up region for photons. The bolus material is made of two components that are kneaded together, directly applied on patient's skin, under a thermoplastic immobilization mask, molded into the desired shape during simulation. A Computed Tomography (CT)‐scan is then acquired for treatment planning with the bolus in place.

The use of highly dense materials, however, necessitates more stringent dosimetric validation within the treatment planning system (TPS), as dose calculations near heterogeneous interfaces present challenging computational conditions^7^ Traditional superposition/convolution algorithms (e.g., Analytical Anisotropic Algorithm (AAA)) rely on empirical approximations and struggle with electron disequilibrium near interfaces.^8^ Conversely, advanced dose calculation algorithms like Acuros XB (AXB) solve the Linear Boltzmann Transport Equation (LBTE) based on theoretical interaction cross sections, approaching the accuracy levels previously exclusive to Monte Carlo techniques.^8^

This study aims to comprehensively evaluate the dosimetric performance of the high‐density bolus when used within the TPS using AXB and AAA algorithms, and alongside to assessacute skin toxicity observed in a clinical cohort.

## 2 MATERIALS AND METHODS

### 2.1 Dosimetric validation

The dose calculation accuracy was evaluated for both photon dose calculation algorithms available in the Eclipse (V18) Treatment Planning System (TPS) (Varian Medical Systems (Palo Alto, CA)): AAA (Analytitical Anisitropic Algorithm)^9^, ^10^ and Acuros XB (AXB).^8^

A CT‐scan was acquired for every setup. The experimental setup is described below, simulating the actual clinical use. The high‐density bolus eXaskin® was simply included into the external contour (not individually delineated) its density or material was not enforced to comply with the vendor specified mass density but left intact and therefore represented by Hounsfield Units (HU) derived from CT data, ranging from 850 and 1000 HU. Measurements were performed using 6 megavoltage (MV) flattening‐filter‐free (FFF) photon beams on both Varian Halcyon and Varian TrueBeam systems. Absolute dose and percentage depth dose (PDD) measurements were performed exclusively on the Halcyon system using a 6 MV FFF beam. In contrast, measurements assessing the impact of air gaps between the bolus and the surface were performed exclusively on the TrueBeam system. Each platform was therefore used for a specific experimental purpose according to platforms availabilities.

**FIGURE 2:**
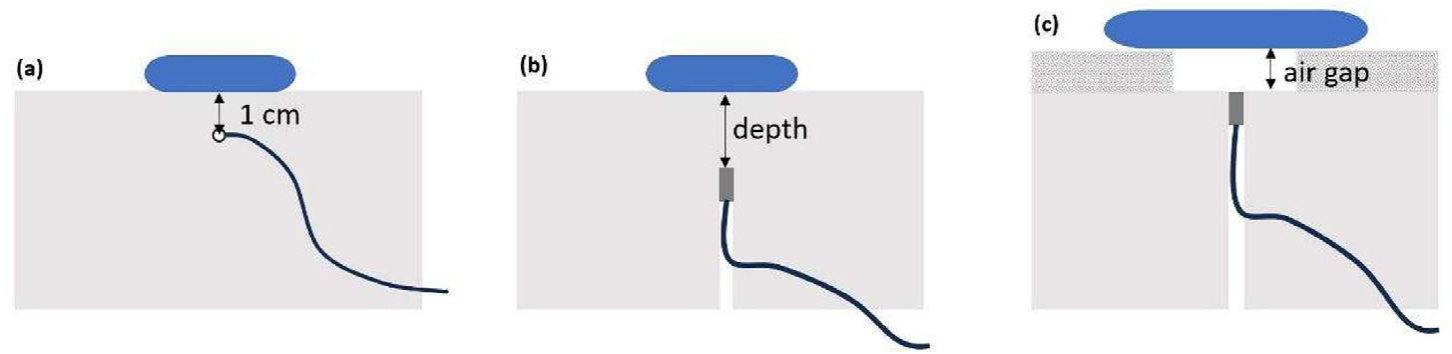
Experimental setup for dosimetric validation of dose calculation: (a) absolute point dose measurement at 1.0 cm beyond the bolus using a Semiflex ionization chamber; (b) depth dose measurement using a microDiamond detector (measurement depth ranging from 0.1 to 6.1 cm); (c) measurement setup to evaluate the impact of bolus–skin air gaps (ranging from 0.5 to 2.0 cm).

Firstly, we simulated the ideal situation in which the bolus is perfectly conforming to patient anatomy and in contact with the skin surface. To assess the accuracy with which of the dose calculation algorithm represents the skin dose beyond the bolus, a 1.0 cm thick bolus was placed directly on solid water slabs (Solid Water®HE Sun Nuclear) and the absolute dose as measured for a 10.0 × 10.0 cm^2^ field (100 Monitor Units (MU)) at a depth of 1.0 cm beyond the bolus (i.e. at a total water equivalent depth of 2.5 cm) through use of a PTW Semiflex 0.3 cc ionization chamber (Figure 2a). To ensure accurate position, the ion chamber was placed with its geometric center at isocenter, corresponding to a source‐to‐surface distance of 99 cm (without bolus). Secondly, we measured the depth dose behavior beyond the bolus. For this purpose, we made a solid water block through which a vertical hole was drilled to accommodate the PTW microDiamond in a vertical position (Figure 2b). Depth dose measurements were performed by incrementally increasing the thickness of solid water above the microDiamond detector, while decreasing the treatment couch so as to maintain a constant source‐to‐surface distance of 99 cm. The bolus was always repositioned on top of the solid water. Taking into account the 0.1 cm intrinsic water equivalent build‐up thickness of the microDiamond, Percentage depth dose (PDD) data were obtained over a range of 0.1–6.1 cm. The relative microDiamond measurements were subsequently rescaled to absolute dose by means of the absolute point dose measurement with the ion chamber. The measured PDDs were compared to the TPS calculation (Eclipse v18, Varian©).

Finally, to assess possible dose loss to the skin (target volume) due to air gaps between bolus and skin, we measured the dose at 0.1 cm depth (i.e. at the intrinsic water equivalent depth of the microDiamond) with increasing air cavity between skin and bolus. We used the same setup as for the PDDs, but instead of placing the bolus directly onto the phantom, we cut a large central hole in thin styrofoam slabs and used these to support the bolus while creating air gaps of 0.5 cm, 1.0 cm, and 2.0 cm between the 1.0 cm thick bolus and the surface of the solid water (Figure 2c). The possible skin underdosage was assessed for different static field sizes (from 3.0 × 3.0 to 10.0 × 10.0 cm^2^) as well as for 0.4,0.6 and 2.0 cm wide dynamic sweeping gap (Swp) MLC (multi‐leaf collimator) deliveries, creating a total irradiated area of 10.0 × 10.0 cm^2^.

### 2.2 Clinical analysis

The retrospective clinical analysis included data from all patients treated with the high‐density Bolus for skin cancers (adjuvant or definitive irradiation) at a single institution between October 2023 and March 2025. Acute skin toxicity (Dermatitis) was graded using the Common Terminology Criteria for Adverse Events (CTCAE) Version 5.0 scale. Local recurrence, defined as relapse in the irradiation field was also reported at last follow up.^11^ Descriptive parameters are expressed as number (%) and median (interquartile 25th‐75th percentiles), unless stated otherwise. Differences between measured and calculated doses are reported with mean, standard and maximal deviation.

## 3 RESULTS

### 3.1 Dosimetric validation

**FIGURE 3:**
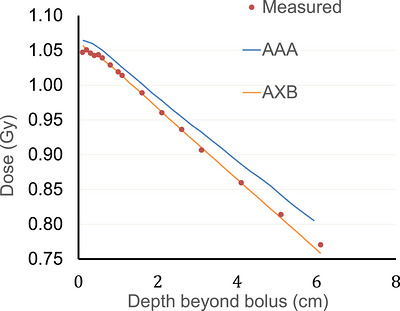
Measured (symbols) and calculated (AAA and AXB) depth dose profiles beyond the bolus.

The comparison between measured and calculated dose beyond the bolus is represented in Figure 3. A good agreement between measured and calculated doses was observed at all depths for both dose calculation algorithms. For AXB, the mean dose difference for all depths is −0.1%, with a standard deviation of 0.5%, and a maximum deviation of 1.50% observed at 6 cm depth. In comparison, AAA showed a mean deviation of 1.70%, with a standard deviation of 0.8% and a maximum deviation of 3.4%.

Table 1 displays the loss of skin dose (dose at 0.1 cm depth) as a result of air gaps between the bolus and the skin. For the 3.0 × 3.0 cm^2^ static field, an air gap of 1.0 cm gives rise to a skin dose loss of more than 5.0%, rapidly escalating to as much as 20.0% for a 2.0 cm gap. The skin dose loss depends on the static field size. For large field size differences remain within 3% of the expected skin dose without any air gaps, as long as the air gap remained below 1 cm. While the dose loss due to a 1.5 cm air gap could still be considered within clinical tolerance for large fields (> 7.0 × 7.0 cm^2^), a 2.0 cm air gap nearly always results in deviations of more than 3.0%.

**TABLE 1:** Relative skin dose loss^c^ (%) at 0.1 cm depth as a function of bolus–skin air gaps thickness for static MLC^a^ fields and dynamic sweeping gap^b^ deliveries.

	Bolus‐skin air gaps (cm)			
Fields	0.5 cm	1 cm	1.5 cm	2 cm
3.0 × 3.0 cm^2^	−2.8%	−5.9%	−12.7%	−21.4%
5.0 × 5.0 cm^2^	−1.4%	−2.6%	−5.3%	−10.2%
7.0 × 7.0 cm^2^	−1.1%	−1.9%	−3.3%	−6.0%
10.0 × 10.0 cm^2^	−0.9%	−1.4%	−2.4%	−3.9%
Sweeping gap 4.0 mm	−0.7%	−1.9%	−2.6%	−4.9%
Sweeping gap 6.0 mm	−0.6%	−1.5%	−1.8%	−3.2%
Sweeping gap 20.0 mm	−0.3%	−1.0%	−1.4%	−2.6%

^a^ MLC: multileaf collimator.

^b^ Sweeping gap deliveries correspond to dynamic sliding window irradiation with gap sizes of 4.0, 6.0, and 20.0 mm.

^c^ Values represent the percentage reduction in measured dose at 0.1 cm depth relative to the reference condition without bolus–skin air gaps.

Considering that, in the case of air gaps, the reduction in skin dose depends on the size of the static field, the sweeping gap deliveries aimed to investigate the impact of MLC segment size on skin dose during dynamic delivery. The sliding window deliveries with 0.4 × 10.0, 0.6 × 10.0, and 2.0 × 10.0 cm^2^ slits display a dose loss that is near‐identical to the results observed in the 10.0 × 10.0 cm^2^ static field. Consequently, it shows that the dose loss is independent of the individual MLC segment size and determined solely by the integral dose delivered.

### 3.2 Clinical outcomes and acute toxicity

The retrospective analysis included data from a cohort of 55 patients (median age: 81 years [76–88]) treated in a single institution from October 2023 to March 2025.

The median biological equivalent dose (BED_10_) delivered was 62.4 Gy [62.4–81.9 Gy]. The main doses schedules used were 48 Gy/16 Fr/3 fractions per week (*n* = 19, 34%) for adjuvant irradiation and 63 Gy/21 fractions/ 3 fractions per week (*n* = 19, 34%) per week for exclusive irradiation or R1/R2 resections. The median dose delivered was 50 Gy in [48–63 Gy] in 3 Gy fraction [2.67–3 Gy].

The acute skin toxicity profile (Dermatitis, CTCAE v5.0^10^) remains low: 50% of the patients exhibit grade 0‐I radiodermatitis (*n* = 27/55), 46% grade II radiodermatitis (*n* = 26/55), and only 4% grade III (*n* = 2/55). Results are presented in Table 2. No treatment had to be stopped due to toxicity.

**TABLE 2:** Distribution of acute radiation‐induced dermatitis according to CTCAE v5.0 in the clinical cohort treated with the high‐density bolus (*N* = 55).

CTCAE grade (Dermatitis)	*N*	Percentage	CTCAE v5.0 definition
Grade 0	0	0.0%	Asymptomatic / intervention not indicated
Grade 1	27	49.1%	Faint erythema or dry desquamation
Grade 2	26	47.3%	Moderate erythema; patchy moist desquamation, mostly confined to skin folds and creases; moderate edema
Grade 3	2	3.6%	Moist desquamation in areas other than skin folds and creases; bleeding induced by minor trauma or abrasion
Grade 4	0	0.0%	Life‐threatening consequences; skin necrosis or ulceration of full thickness dermis; spontaneous bleeding from involved site; skin graft indicated.

Median follow up was 5.7 months [3.9–11.7]. Three patients (5.4%) exhibited local failure with a mean delay of 4 months after start of irradiation.

## 4 DISCUSSION

This study showed that when a high‐density bolus material is used, a thickness of 1.0 cm is sufficient to overcome the 1.3 cm build‐up region of a 6FFF photon beam which is the lowest and the most used photon energy worldwide. Comparison between measurements and dose calculations beyond the bolus show acceptable (< 3.5%) results when using the AAA dose calculation algorithm but with better agreement (< 1.5%) when using the AXB algorithm. A slightly larger discrepancy observed at the first measurement point could be explained by accumulated uncertainties in the build‐up region. In dose calculation, both the calculation grid resolution and the approximation of electron contamination models have their largest impact near the surface. On the measurement side, the lack of charged particle equilibrium in the build‐up region is known to affect detector response and measurement accuracy. Furthermore, Hounsfield units derived from CT data were sufficient for dose calculation, with good agreement observed between measured doses and AXB calculations. Given the HU values attributed to the bolus on CT (800 to 1000 HU), AXB automatically assigns bone of moderate density to the bolus during calculation.

The impact of air gaps between the bolus and the skin should not be underestimated. Deleterious effects of air gaps are more important for small static fields. Dynamic deliveries with small MLC segments illustrate that the dose loss depends on the integral dose and not on the individual segment size. While the primary dosimetric validation was performed using static open fields, additional measurements using dynamic sweeping gap deliveries were conducted to better approximate modulated treatment conditions such as IMRT or VMAT. These dynamic deliveries, based on sliding window irradiation, allow partial simulation of the temporal and spatial dose modulation inherent to these techniques. The observed dosimetric behavior under these conditions was consistent with that of static fields, indicating that the impact of air gaps is primarily driven by the integral delivered dose rather than by individual segment characteristics. Although these simplified dynamic deliveries do not fully reproduce the complexity of clinical IMRT or VMAT treatments, the results support the applicability of our findings to modulated delivery techniques. Therefore, the size of the actual lesion (PTV) can be used as a good indication to estimate the dosimetric deleterious impact of possible air gaps. Importantly, the impact of air gaps appears to be strongly dependent on the treated volume. While small target volumes may be more sensitive to local dosimetric perturbations induced by air cavities, larger treated surfaces tend to mitigate this effect due to increased lateral scatter and dose averaging. For small lesions (< 5.0 cm), skin dose losses can exceed 10% in the presence of a 2.0 cm air gap. Ideally, the bolus should therefore be made as conform as possible and in contact with the skin over the entire target volume. This suggests that the clinical relevance of air gaps should not be considered uniformly, but rather interpreted in the context of the target size. In small volumes even limited air gaps may lead to non‐negligible underdosage and therefore require careful consideration. On the opposite, for large volumes, the dosimetric impact is reduced and larger airgaps could be tolerated. Even if for larger volumes, the dose loss is less substantial, however, one should still aim to minimize bolus–skin air gaps as much as possible, as even small air gaps have been shown to reduce surface dose in photon beam radiotherapy, with the magnitude of this effect increasing with gap size and depending on field size and beam incidence.^6^ From a practical endpoint, these findings indicate that a new simulation CT in the presence of air gaps may not always be necessary, particularly for large treated areas. A more pragmatic, case‐by‐case approach could therefore be considered. The high malleability of the high‐density bolus material presents an important advantage when irregular areas (nose, ears, extremities) need to be treated. Furthermore, the bolus was applied directly on the skin rather than over the thermoplastic immobilization mask, thereby reducing the probability of unintended air gaps.

The clinical results confirmed the safety profile of our strategy, demonstrating that its use does not lead to an unexpected high rate of severe acute skin reactions, even for treatments delivered to anatomically complex areas (e.g., ear, nose) in an elderly cohort (median age 81 years). The low rate of Grade III and IV dermatitis (3% with no grade IV toxicities) suggests that the dose accuracy achieved by combining AXB planning with the conforming bolus successfully targets the superficial lesion without any overdosage to the surrounding skin tissue.

From a broader perspective, the technique appears to be a clinically relevant and robust solution for optimizing skin dose in external beam radiotherapy on modern platforms. It could be a safe alternative to advanced modalities such as proton therapy, for whom ballistic is superior and the lack of bolus effect is advantageous. However proton therapy represents a substantial technological and financial investment, limiting its accessibility to a restricted number of centers.^12^ Conversely, kilovoltage‐based skin treatment approaches (e.g., dedicated kV devices) remain gold standard for very superficial and small tumors but are not suitable for the treatment of larger or deeper target volumes.^13^ Finally, three‐dimensional printed bolus solutions provide excellent conformity to patient anatomy; however, they require non‐negligible design and manufacturing time, specific expertise, and dedicated material resources.^14^ In this context, the proposed solution represents a balanced compromise between dosimetric effectiveness, clinical feasibility, economic cost and integration into routine workflows.

## 5 CONCLUSION

This study assessed the dosimetric performance of a high‐density, high malleable bolus to compensate for the skin‐sparing effect of megavoltage photon beams in the treatment of superficial skin cancer on modern radiotherapy platforms. A 1 cm thickness bolus effectively addresses the skin dose deficit when using 6FFF photon beams. Acuros XB was the preferred calculation algorithm due to its superior accuracy in modeling high‐density/soft‐tissue interfaces. To avoid unintentional skin dose loss due to air cavities between bolus and skin, bolus–skin air gaps should be minimized as much as possible The impact of air gap is however lower when Dynamic delivery is used or when large target volumes are used. Combined with reassuring clinical toxicity data, high‐density and malleable bolus represents a viable and accessible solution for ensuring accurate and conformal dose delivery in superficial tumors.

## AUTHOR CONTRIBUTIONS

Jonathan Dadoun conceptualized the study, designed the action plan, performed the measurements, coordinated the project, drafted the manuscript, and submitted the work. Ann Van Esch contributed to the design of the action plan, provided measurement equipment, and participated in writing and revising the measurement section of the manuscript. Luc Ollivier contributed to the writing and revision of the clinical section. Carine Lefrancois‐Clement collected and analyzed cutaneous toxicity data from the study patients. Erwan Luce assisted with measurements and generated radiotherapy treatment plans delivered to the patient cohort. Alexis Thelie, Nesrine Zemman, Nassima Delhoumi, and Andrea Feuillet contributed to the development of radiotherapy treatment plans for the study cohort. Romain Mallet, Renata Pereira, Laurent Martin, and Paul Lesueur, as physicians were responsible for clinical management and follow‐up of the patients. Noemie Litrowski, Paul Gangloff, and Christophe Moure, contributed to patient care and reviewed the manuscript. Paul Lesueur also supervised the study and contributed to the overall critical revision of the manuscript. All authors reviewed and approved the final manuscript.

## CONFLICT OF INTEREST STATEMENT

The authors declare no conflicts of interest.

## ETHICAL APPROVAL

This study was approved by French Ethics Committees and the National Commission on informatics and Liberties (MR003 Methodology). An information letter was sent to patients still alive at time of data collection. This study adhered to the Declaration of Helsinki.

## Data Availability

The data presented in this study are available from the corresponding author upon reasonable request.
